# COVID-19 Concerns, Depression and Loneliness in Middle-Age and Older Adults

**DOI:** 10.1093/geroni/igab046.2745

**Published:** 2021-12-17

**Authors:** Angela Curl, Katie Wolf

**Affiliations:** Miami University, Oxford, Ohio, United States

## Abstract

The coronavirus pandemic forced many people to restrict their activities and social interactions out of fear and due to local health regulations. This study examined whether one’s self-reported level of concern related to COVID-19 was associated with loneliness and depressive symptoms. Using early release 2020 data from the Health and Retirement Study (N=2,759 adults over age 50), we conducted ordinary least-squares and logistic regressions, controlling for age, gender, education, marital status, self-rated health, and exercise. Higher levels of self-reported concern about the coronavirus pandemic were associated with more depressive symptoms (B=.05, p<.01) and increased odds of being lonely (OR=1.05, p<.01). Female sex, lower education, not being married, worse self-rated health, and lack of exercise were associated with more depressive symptoms and higher odds of being lonely, while older age was associated with lower depression but higher odds of loneliness. These results suggest that mental health assessments should include measures specifically asking about COVID-19 concerns and experiences (e.g., COVID-19 diagnosis, death of close friends or family due to COVID-19, unable to attend important events). The pandemic has raised public awareness of the negative consequences of social isolation and acted to destigmatize mental illness, and this could encourage middle-aged and older adults to seek professional help for depression.

